# Computational and Experimental ^1^H-NMR Study of Hydrated Mg-Based Minerals

**DOI:** 10.3390/molecules25040933

**Published:** 2020-02-19

**Authors:** Eric G. Sorte, Jessica M. Rimsza, Todd M. Alam

**Affiliations:** 1 Department of Organic Material Sciences, Sandia National Laboratories, Albuquerque, NM 87185, USA; egsorte@sandia.gov; 2 Department of Geochemistry, Sandia National Laboratories, Albuquerque, NM 87185, USA; jrimsza@sandia.gov

**Keywords:** ^1^H-NMR, chemical shift, magnesium oxide, magnesium minerals, hydroxylated, DFT, GIPAW

## Abstract

Magnesium oxide (MgO) can convert to different magnesium-containing compounds depending on exposure and environmental conditions. Many MgO-based phases contain hydrated species allowing ^1^H-nuclear magnetic resonance (NMR) spectroscopy to be used in the characterization and quantification of proton-containing phases; however, surprisingly limited examples have been reported. Here, ^1^H-magic angle spinning (MAS) NMR spectra of select Mg-based minerals are presented and assigned. These experimental results are combined with computational NMR density functional theory (DFT) periodic calculations to calibrate the predicted chemical shielding results. This correlation is then used to predict the NMR shielding for a series of different MgO hydroxide, magnesium chloride hydrate, magnesium perchlorate, and magnesium cement compounds to aid in the future assignment of ^1^H-NMR spectra for complex Mg phases.

## 1. Introduction

Magnesium oxide (MgO) is used in many engineering applications because of its unique material properties. For example, its high thermal conductivity contributes to its use as a stable refractory material [1], and its structure makes it an ideal basic oxide model [2,3]. MgO has also been used for construction [4,5], optics [6,7], medicine [8,9], fertilizers [10], electronics [11,12], catalysis [13], chemical warfare agent decontamination [14], high temperature super conductors [15], hydrogen storage [16,17], and nuclear waste storage [18,19]. For many of these applications, the performance of MgO depends on property reproducibility that may be impacted by impurities or defects formed from the conversion of MgO to other magnesium-containing phases due to environmental exposure. In many cases, the resulting MgO conversion phases are disordered or amorphous in nature, making their characterization by scattering techniques challenging. For example, our interest is the identification of protonated impurities in MgO [20]: a material that is currently used as an engineered barrier for carbon remediation at the DOE Waste Isolation Pilot Plant (WIPP) located near Carlsbad, New Mexico.

Nuclear magnetic resonance (NMR) spectroscopy is a powerful technique for characterizing disordered solids where diffraction methods may fail. Despite its usefulness, only a few publications have reported solid state ^1^H-NMR spectra for MgO-based compounds [3,20,21,22,23,24,25,26]. Previous studies noted that the ^1^H chemical shifts in basic environments are shifted to lower frequencies (~0 ppm), while acidic environments exhibit higher frequency chemical shifts (~4.5 ppm) [27]. In a previous study, at least six different proton environments were observed (but not fully assigned) in nominally pure MgO between 1.2 and −1.8 ppm [3]. Extensive NMR characterization (including ^1^H-NMR) of hydrous ringwoodite (γ-Mg_2_SiO_4_) [28], hydroxyl-clinohumite (4Mg_2_SiO_4_·Mg(OH)_2_) [29], and hydrous wadsleyite (β-Mg_2_SiO_4_) have also been published [30,31,32]. Most computational NMR efforts on MgO-related phases have focused on ^25^Mg and ^17^O, but for low concentration impurity or defect phases these nuclei can be difficult to address experimentally. There has been at least one thorough computational effort using density functional theory (DFT) to model OH groups on irregularities in MgO surface states [2]. In this work, we report the ^1^H magic angle spinning (MAS) NMR spectra for a set of MgO-based minerals, which are reaction products of MgO with different environmental constituents. Using computational NMR methods, the correlation between the predicted NMR chemical shielding and the experimental NMR chemical shift is developed to allow ^1^H-NMR chemical shift referencing in Mg oxide compounds. These computational NMR methods are then extended to develop a starting database of ^1^H-NMR chemical shifts of proton-containing Mg phases not readily isolated or available.

## 2. Results and Discussion

**Scheme 1:**
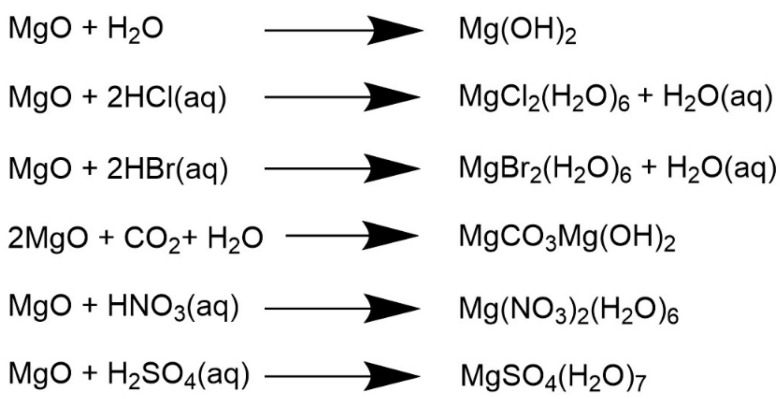
Examples of different MgO conversion reactions.

MgO can convert to a variety of different compounds, including naturally occurring minerals, by incorporating new coordinating species during environmental exposure. These include hydroxylated, hydrated, nitrated, and sulfonated magnesium phases derived from either MgO or MgCO_3_ through simple conversion reactions (Scheme 1). These new Mg-containing species may act as impurities or defects within the original bulk materials, which could ultimately impact the targeted material performance.

### 2.1. Model Hydroxylated and Hydrated Magnesium Compounds

**Figure 1:**
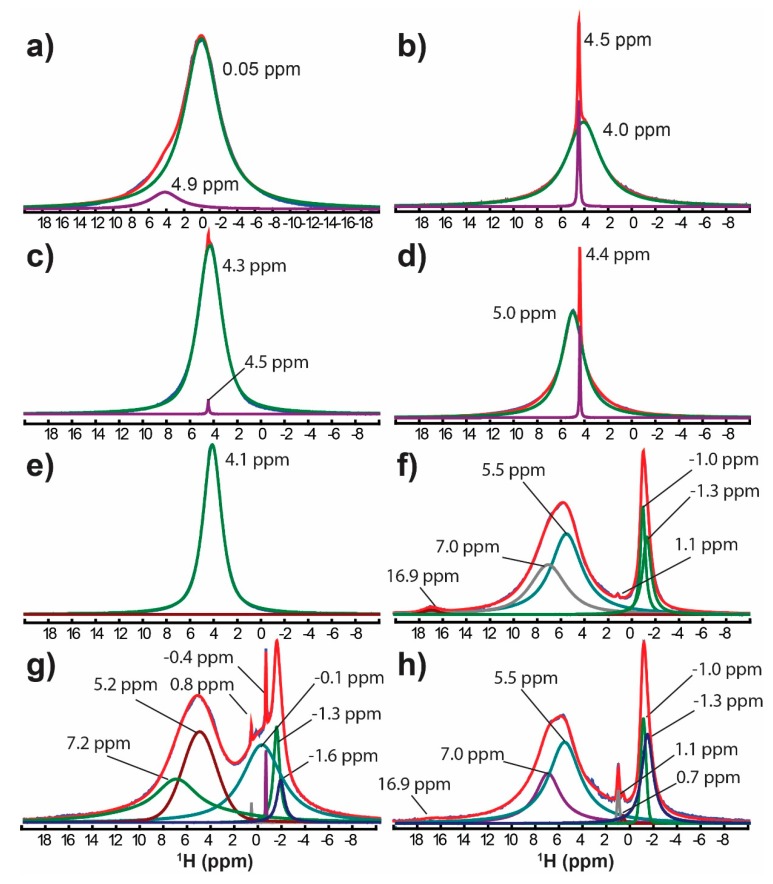
Deconvolution of the ^1^H- magic angle spinning (MAS) nuclear magnetic resonance (NMR) spectra for (**a**) brucite, Mg(OH)_2_, (**b**) nitromagnesite, Mg(NO_3_)_2_·6H_2_O, (**c**) bischofite, MgCl_2_·6H_2_O, (**d**) epsomite, MgSO_4_·7H_2_O, (**e**) magnesium bromide, MgBr_2_·6H_2_O, (**f**) hydromagnesite, 4MgCO_3_·Mg(OH)_2_·4H_2_O, (**g**) artinite, MgCO_3_·Mg(OH)_2_·3H_2_O, and (**h**) dypingite, 4MgCO_3_·Mg(OH)_2_·5H_2_O.

The ^1^H-MAS-NMR spectra for a series of different Mg minerals is shown in Figure 1 with the experimental chemical shifts summarized in Table 1. Brucite, Mg(OH)_2_, is formed from the absorption of water by MgO and is a common hydrogen-containing phase observed in environmentally exposed materials [20]. The ^1^H NMR spectrum of Mg(OH)_2_ is shown in Figure 1a. The dominant resonance at δ = +0.05 ppm (93%) is assigned to the hydroxyl proton in Mg(OH)_2_, while the minor resonance at δ = +4.9 ppm (7%) is attributed to a water containing phase in this material. In cement applications, Mg(OH)_2_ may cause structural expansions inducing stress, cracking, and failure in concrete and is therefore important to identify [5]. Nitromagnesite, MgNO_3_·6H_2_O, is a highly water-soluble magnesium salt and can be formed by mixing magnesium sulfate with calcium nitrate, or by simply exposing MgO, Mg(OH)_2_, or magnesium carbonate (MgCO_3_) to nitric acid [33]. The ^1^H-MAS-NMR spectrum of nitromagnesite is shown in Figure 1b, with the major resonance at δ = +4.0 ppm assigned to the bound water protons, while the small sharp resonance at δ = +4.5 ppm results from mobile surface adsorbed water in this sample. Heating nitromagnesite leads to decomposition of the salt rather than dehydrating it, producing undesirable NO_3_. Bischofite, MgCl_2_·6H_2_O, can be formed by the neutralization of dilute HCl by MgO. The ^1^H-NMR spectrum of bischofite is shown in Figure 1c with the bound water protons observed at δ = +4.3 ppm. The formation of bischofite phases may impact the refractory application of MgO, since MgCl_2_ has a melting point (987 K) almost three times lower than MgO [33]. Epsomite, MgSO_4_·7H_2_O, can be formed by the reaction of MgO with dilute sulfuric acid to produce MgSO_4_, which is stable in the heptahydrate form up to 48 °C in air and high relative humidity (RH). Above this temperature (or in low RH), dehydration occurs to form the hexahydrate, until finally heating between 200–300 °C produces an anhydrous phase [33]. The loss of water during dehydration changes the crystal structure (i.e., the heptahydrate’s orthorhombic structure evolves to a monoclinic structure in the hexahydrate). The ^1^H-NMR spectrum of epsomite is shown in Figure 1d, with the bound water proton environment assigned to the δ = +5.0 ppm resonance. A mobile surface adsorbed water environment is observed at δ = +4.4 ppm. Magnesium bromide, MgBr_2_·6H_2_O, is produced by reacting MgCO_3_ with HBr and can form both anhydrous and hexahydrate forms [33]. The ^1^H-NMR spectrum of the hexahydrate phase is shown in Figure 1d, with the water proton resonance at δ = +4.1 ppm. Magnesium carbonate can exist in many forms, including multiple minerals. The most common is the anhydrous salt magnesite MgCO_3_. The ^1^H-NMR spectra of several common hydrated magnesium carbonate salts are shown, including hydromagnesite (4MgCO_3_·Mg(OH)_2_·4H_2_O, Figure 1f, artinite (MgCO_3_·Mg(OH)_2_·3H_2_O, Figure 1g, and dypingite (4MgCO_3_·Mg(OH)_2_·5H_2_O, Figure 1h. These NMR spectra show a hydroxyl proton environment at *δ* ~ −1.3 ppm along with multiple water proton species between +5.0 and +7.0 ppm. The multiple overlapping water proton environments are further characterized using two-dimensional (2D) double quantum (DQ)-single quantum (SQ) NMR correlation experiments discussed in a later section. The hydroxyl resonance was asymmetric such that two different hydroxyl environments were included to improve the deconvolution of this spectral region. There is no clear resolution of the different hydroxyl environment, though the 2D DQ-SQ NMR correlation experiments also supports the argument of overlapping hydroxyl species. For artinite (MgCO_3_·Mg(OH)_2_·3H_2_O, an additional hydroxyl environment at δ = −0.1 ppm was also observed and is clearly identified in the 2D DQ-SQ correlation experiments (see below). Two different commercial sources of dypingite were analyzed and produced equivalent NMR spectra (Supplementary Materials, Figure S1, electronic supplementary information ESI). An acidic proton at δ = 16.9 ppm was seen for hydromagnesite and dypingite (<1%). Minor unidentified impurity phases between δ = +0.7 and +1.1 ppm were also present [30]. While the NMR spectra for these three carbonates phases are similar, the ratio of the hydroxyl and hydrating water proton concentration might also be used to support the proposed structure. Artinite (MgCO_3_·Mg(OH)_2_·3H_2_O) has the largest [OH]/[H_2_O] ratio of 2/6 (25%/75%) and compares to the 22%/73% ratio observed experimentally (Figure S3, ESI). Hydromagnesite (4MgCO_3_·Mg(OH)_2_·4H_2_O) should have a [OH]/[H_2_O] ratio of 2/8 = (20%/80%), which is consistent with the 19%/81% ratio measured (Figure S3). Dypingite (4MgCO_3_·Mg(OH)_2_·5H_2_O has the lowest [OH]/[H_2_O] ratio of 2/10 (16.7%/83.3%), comparable to the 18%/82% observed. The error inherent in the deconvolution of multiple overlapping resonances, along with the error resulting from differential spin-spin (T_2_) relaxation on the water and hydroxyl protons and the need to extrapolate to zero echo time (ESI) make assignments based simply on the [OH]/H_2_O] intensity ratio difficult. Currently, there are no crystal structures available for these hydrated magnesium carbonate phases, but the NMR results reveal the different structural motifs and relative concentrations present.

The experimental NMR results for all the compounds listed in Table 1 represent only two major types of proton environments (hydroxyl and coordinating water) with subtle differences in the coordination environments giving rise to small changes in the ^1^H-NMR chemical shifts. The very small variations further support the argument that computational NMR efforts are needed to help characterize proton containing magnesium phases (Section 2.2).

#### Two-Dimensional Double Quantum NMR

**Figure 2:**
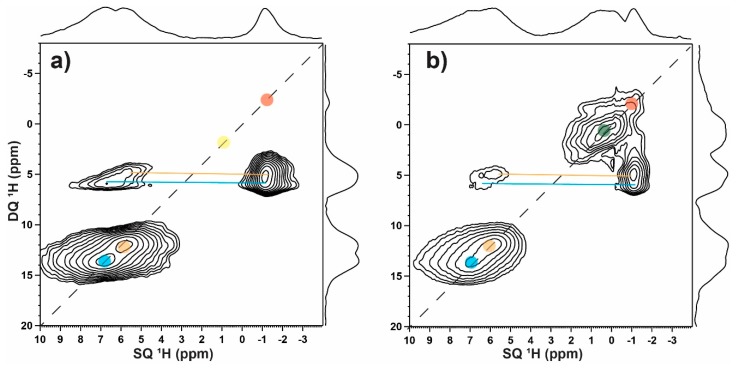
2D double quantum (DQ)-single quantum (SQ) ^1^H-MAS-NMR correlation spectra for (**a**) hydromagnesite, 4MgCO_3_·Mg(OH)_2_·4H_2_O and (**b**) artinite, MgCO_3_·Mg(OH)_2_·3H_2_O. The auto-correlation peaks between protons in the same environment (chemical shift) are identified by solid circles, and the colored lines identify correlation between different proton environments.

To further characterize the multiple proton environments in the carbonate series 4MgCO_3_·Mg(OH)_2_·4H_2_O, MgCO_3_·Mg(OH)_2_·3H_2_O, and 4MgCO_3_·Mg(OH)_2_·5H_2_O, 2D DQ-SQ NMR correlation spectra were obtained. As an example, Figure 2a is the 2D DQ-SQ NMR spectrum for hydromagnesite (4MgCO_3_·Mg(OH)_2_·4H_2_O), while Figure 2b in the DQ-SQ spectrum for artinitie (MgCO_3_·Mg(OH)_2_·3H_2_O). The 2D DQ-SQ NMR correlation spectrum for dypingite (4MgCO_3_·Mg(OH)_2_·5H_2_O) was very similar to the hydromagnesite spectrum (Figure 2a) and is provided in Figure S2 for comparison (ESI). In these 2D-NMR correlation experiments, cross peaks are observed at spectral frequencies for protons that are spatially near each other with significant ^1^H-^1^H dipolar coupling to produce the DQ coherence. Cross peaks on the diagonal (filled circles) are auto-correlation resonances and arise from dipolar coupling (spatially near each other) between equivalent ^1^H environments. Off diagonal cross peaks (solid lines) result from dipolar coupling between two different ^1^H environments. The 2D DQ-SQ NMR correlation spectra show multiple cross peaks and allow for improved spectral separation between the different ^1^H environments.

For hydromagnesite (Figure 2a), two water resonances are more clearly observed at δ = +5.5 ppm and + 7.0 ppm, with both water environments having auto-correlation peaks (solid blue and orange circles). This means that the water protons are spatially close to other water protons in equivalent environments (as expected). There is also some off-diagonal intensity between these two water resonances, suggesting that the water environments are spatially near each other but are not in rapid exchange on the NMR time scale (chemical shift difference). The water and hydroxyl protons (δ = −1.2 ppm) also reveal a strong off-diagonal correlation peak (solid blue and orange lines), confirming that the water and hydroxyl proton environments are spatially near each other (i.e., dipolar coupled). Note that the hydroxyl cross peak reveals slightly different hydroxyl chemical shifts (minor slope in DQ dimension) but is not resolved in the 2D projections or the one dimensional (1D) ^1^H-MAS-NMR spectra (Figure 1f). The hydroxyl protons (δ ~ −1.3 ppm) also show a weak auto-correlation peak (solid red circle) but can be seen in the DQ projection, demonstrating that these hydroxyl protons have limited spatial interactions with equivalent hydroxyl proton environments. Finally, the small sharp resonance at δ ~ +1 ppm shows a vanishing weak auto-correlation peak (yellow solid circle) but reveals no other cross peaks. This means that this proton environment is spatially near similar protons but is spatially isolated from all the other protons in these materials. A minor isolated unidentified phase has been observed in previous studies on Mg silicates [30,32], while a δ = +1.1 ppm resonance has been reported for hydrous Chondrodite, Mg_5_Si_2_O_8_(OH)_2_ [25], as well as Talc, Mg_3_Si_4_O_10_(OH)_2_ [26]. The existence of this auto-correlation peak in the 2D DQ-SQ NMR correlation spectrum also argues that this proton is not simply probe background but is indeed a unique proton containing phase. Similarly, for artinite (Figure 2b), two water environments are observed at δ = 7.1 and δ = + 5.2 ppm with strong auto-correlation peaks (solid blue and orange circles). The off-diagonal cross peaks between the water and hydroxyl protons (δ = −1.4 ppm) are also observed (solid blue and orange lines), confirming these proton environments are spatially near each other. Unique to artinite is the presence of a strong auto-correlation peak neat δ ~0.0 ppm (green circle) arising from isolated hydroxyl protons. The hydroxyl protons are only spatially near equivalent hydroxyl protons (giving rise to an auto-correlation peak) but show no dipolar coupling to water proton environments. This contrasts with the hydromagnesite (Figure 2a) and dypingite (Figure S2) spectra, where the hydroxyl environments are isolated from each other and are instead dipolar coupled to nearby water protons. These 2D ^1^H-DQ-SQ-NMR correlation experiments reveal how it is possible to improve resolution between different proton species as well as probe the spatial arrangement of the different proton environments.

### 2.2. Computational ^1^H-NMR

#### 2.2.1. Chemical Shift Referencing

To compare NMR results, both the experimental and calculated chemical shifts (δ) are defined with respect to the isotropic trace of the chemical shielding tensor $\sigma_{iso}$.

(1) $$\begin{array}{l} \delta^{exp}{=\sigma }_{ref}^{exp}-\;\;\sigma_{iso}^{exp}\\ \delta^{calc}{=\sigma }_{ref}^{calc}-\;\;\sigma_{iso}^{calc} \end{array}$$

where $\sigma_{ref}$ is the chemical shielding tensor for a reference compound. It has been established that the DFT gauge-including projector augmented wave (GIPAW) method (used here) has a linear relationship between the variation in the experimental $\left(\delta^{exp}\right)$ and calculated chemical shifts $\left(\delta^{calc}\right)$ [36], and that $\sigma_{ref}^{exp}$ and $\sigma_{ref}^{calc}$ are not necessarily equivalent. This linear variation is given by

(2) $$\Delta \delta^{exp}=\alpha \Delta \delta^{calc}$$

**Figure 3:**
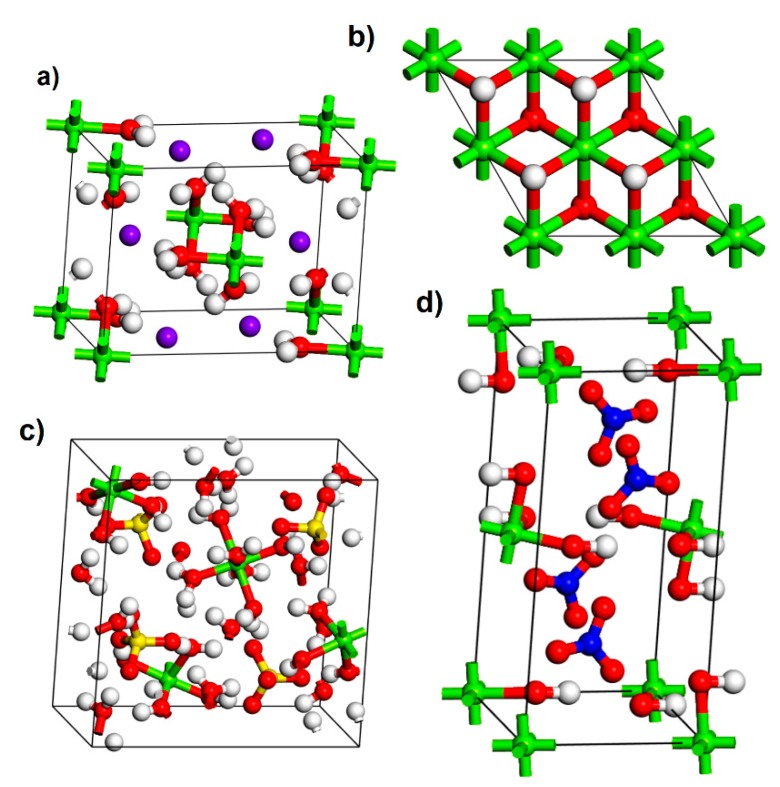
Snapshots of four different MgO-containing crystal structures (**a**) MgCl_2_·6H_2_O, (**b**) Mg(OH)_2_, (**c**) MgSO_4_·7H_2_O, and (**d**) MgNO_3_·6H_2_O. Atom colors: Mg (green), O (red), H (white), Cl (purple), S (yellow), and N (blue).

**Figure 4:**
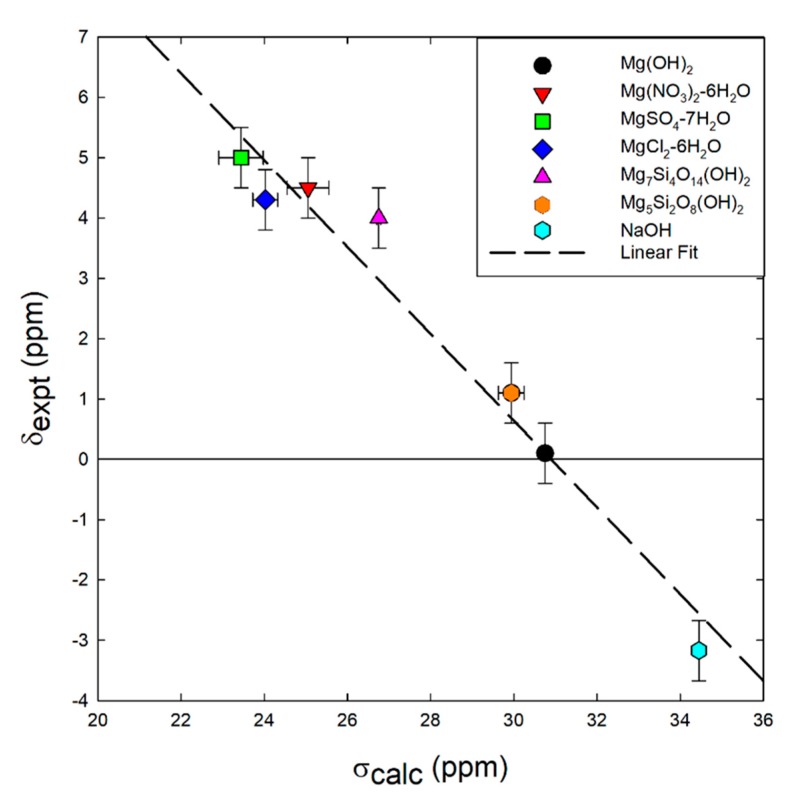
Correlation (R^2^ = 0.9268) between the average computational ^1^H-NMR shielding and experimental chemical shift for select MgO crystalline structures.

This leads to the correlation relationships between the calculated and experimental values for systems where a structure is available

(3) $$\begin{array}{l} \delta^{exp}=\sigma_{ref}^{exp}+\alpha \sigma_{iso}^{calc}\\ \delta^{exp}=\beta +\alpha \sigma_{iso}^{calc} \end{array}$$

with $\beta =\sigma_{ref}^{exp}=\alpha \sigma_{ref}^{calc}$ Because our research interest is the formation of secondary and intermediate phases of MgO during interaction with water [20], we have focused on reference compounds containing both Mg and either OH^-^ or H_2_O components. NaOH was included because, in some applications, exposure to sodium chloride containing solutions (e.g., ground or seawater) may allow for the formation of solid NaOH during the MgO hydration reaction. For calibration of the chemical shift referencing, MgCl_2_·6H_2_O (bischofite), Mg(OH)_2_ (brucite), MgSO_4_·7H_2_O (epsomite), Mg_7_Si_4_O_14_(OH)_2_ (wadsleyite), MgNO_3_·6H_2_O (nitromagnesite), OH-chondrodite (Mg_5_Si_2_O_8_(OH)_2_), and crystalline NaOH were used. See structural snapshots of selected Mg minerals in Figure 3. The experimental ^1^H-MAS-NMR chemical shifts $\delta^{exp}$ along with the computed average chemical shieldings $\sigma_{iso}^{calc}$ are included in Table 1 (bold) and are plotted in Figure 4. Table S1 provides the complete list of predicted ^1^H-NMR chemical shieldings and shifts for the reference compounds. The $\delta^{exp}$ range from −3.2 ppm (NaOH) to +5.0 ppm (MgSO_4_·7H_2_O), while the $\sigma_{iso}^{calc}$ range from +34.5 to +22.9 ppm. Using Equation (3), α = 0.701, $\sigma_{ref}^{exp}$ = 21.52 ppm, and $\sigma_{ref}^{calc}$ = 30.7 ppm. This compares well with the $\sigma_{ref}^{calc}$ of + 31.0 ppm reported based on referencing to the single Mg(OH)_2_ chemical shift [31], and to the +29.76 ppm and +29.59 ppm recently reported for semi- and fully-hydrated wadsleyite [32]. In the future, this chemical shielding correlation could be improved by including experimental NMR for additional compounds with an isolated hydroxyl proton environment (*δ* < 0 ppm).

**Figure 5:**
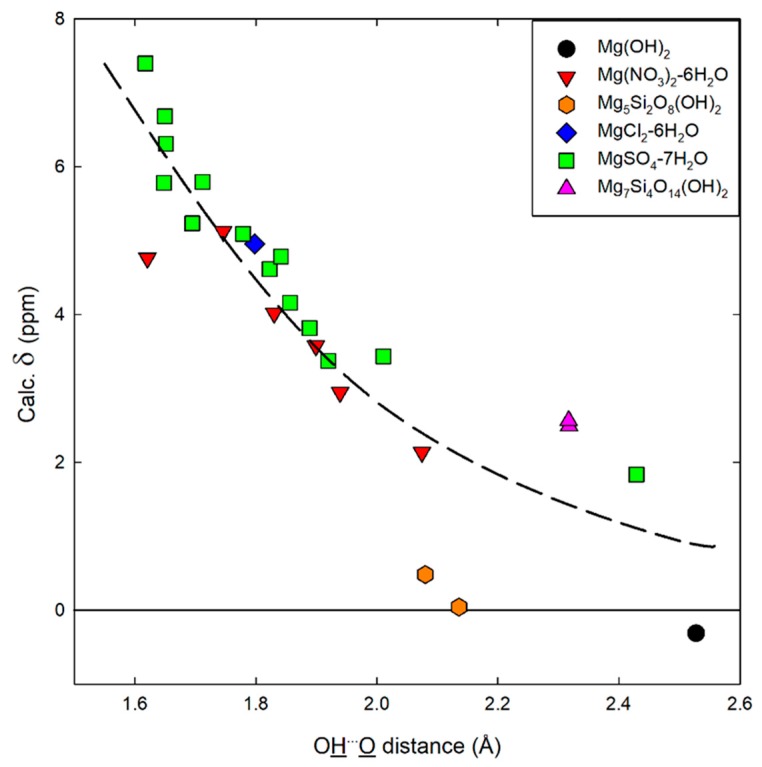
Calculated ^1^H-NMR isotropic chemical shift (δ) values versus OH^…^O hydrogen bond lengths (Å) for refence compounds. The dashed line serves as a guide line to the reader.

Additionally, the selected reference compounds include hydrogen bond lengths from 1.62Å (nitromagnesite and bischofite) to 2.53Å (brucite). The relationship between hydrogen bond length (OH^…^O) and δ has been previously noted by Pourpoint et al. [37] and Xue and Kanzaki [38], with increasing hydrogen bond lengths resulting in a decrease in the calculated δ value. Our results indicate the same trend (Figure 5) and compounds with isolated hydroxyls exhibit lower δ values than those that contain molecular water. Because the referenced compounds include such a large range of hydrogen bonding lengths, the fitting discussed above can be applied to a variety of materials with proton containing phases.

#### 2.2.2. Predicted Chemical Shifts for Hydrogen Containing MgO Phases

In addition to the MgO minerals used for the development of the chemical shift/shielding correlation above (Figure 4), the predicted ^1^H-NMR chemical shielding and chemical shifts of multiple Mg-O-H, Mg-Cl-O-H, and Na-O-H phases are shown in Table 2. These compounds have all been suggested as conversion species of MgO and therefore could be probed by ^1^H-MAS-NMR. Unfortunately, samples of these phases were not readily available, precluding experimental measurements of their chemical shifts. As expected, ^1^H chemical shifts of the Mg hydroxides are predicted to fall into a very narrow range between δ = 0.0 and +0.8 ppm, while the MgCl_2_ hydrate water protons have chemical shifts that are predominantly in the δ = +4.0 to +5.0 range. The ^1^H environments in the Mg perchlorates are predicted to have unique chemical shifts in the δ ~ +3 ppm range. The Mg-cements and layered NaOH compounds are predicted to have very complex and rich chemical shift signatures ranging from δ = −2.3 to 11.6 ppm. The small ^1^H chemical shifts ranges will make it difficult to uniquely identify a given phase based simply on 1D ^1^H-NMR spectra, but 2D NMR correlation experiments will improve the resolution while also providing the means to identify the multiple ^1^H environments arising from a single compound. These results also allow one to quantify the different general proton environments (i.e., hydroxyl, hydrating water, acidic, etc.) that exist in a material. It is hoped that future research into different MgO phases may benefit from these calculated ^1^H-NMR shieldings and shifts for spectral assignment.

## 3. Materials and Methods

### 3.1. Materials

Mg-based compounds were used as-received except where otherwise stated with the purity provided by the vendor. Reagent grade brucite Mg(OH)_2_, (Sigma Aldrich, St. Louis, MO, USA, 95% pure) was acquired in 2011 and had been stored under laboratory conditions. Prior to the NMR analysis, the brucite was dried under vacuum in the presence of P_2_O_5_ for 24 h. Nitromagnesite MgNO_3_·6H_2_O (Fisher Scientific, Waltham, MA, USA, 98% pure), bischofite MgCl_2_·6H_2_O (Sigma Aldrich, 98% pure), epsomite MgSO_4_·7H_2_O (Sigma Aldrich, >98% pure), magnesium bromide MgBr_2_·6H_2_O (Alfa Aesar, Haverhill, MA, USA, >98% pure), hydromagnesite 4MgCO_3_·Mg(OH)_2_·4H_2_O (Alfa Aesar, 99.996% pure), artinite MgCO_3_·Mg(OH)_2_·3H_2_O (Arcos Organics, 98% pure), and dypingite 4MgCO_3_·Mg(OH)_2_·5H_2_O (Sigma Aldrich and Fisher Scientific for vendor comparison, >98% pure) were used as received without further purification.

### 3.2. Experimental NMR

The solid state ^1^H- magic angle spinning (MAS) NMR experiments were performed on a Bruker Avance III spectrometer (Bruker, Billerica, MA, USA) at a proton observation frequency of 600.1 MHz, using a 2.5 mm broadband MAS probe spinning at 30 kHz. Spectra were obtained using a rotor-synchronized Hahn Echo pulse sequence with a 4 s recycle delay and a π/2 pulse length of 2.5 µs. To assure quantitative spectra, the recycle delay was chosen to allow for complete spin lattice (T_1_) relaxation. To correct for the impact of spin-spin relaxation (T_2_) on the Hahn Echo spectra, the signal intensity for the zero-time echo delay (τ = 0) was obtained from the S(τ) versus τ correlations, as shown in Figure S3. For the samples reported here, the impact of differential T_2_ relaxation was most notable when comparing multiple water and hydroxyl environments, such as observed in the Mg carbonate series of materials. Due to frictional heating, the sample temperature was nominally 325 K. The 2D DQ-SQ ^1^H-MAS-NMR correlation spectra were obtained at a 25 kHz spinning speed using an offset compensated back-to-back (BaBa) multiple pulse sequence for excitation and reconversion of the multiple quantum coherences [39], using a single rotor-period rotor period excitation (τ_DQ_ = 40 μs, N_c_ = 1), with phase-sensitive detection in the F1 dimension via the States time-proportional phase incrementation method. ^1^H-NMR chemical shifts were referenced to the secondary standard adamantane (δ = +0.8 ppm) with respect to TMS (δ = 0 ppm). The DMFIT software was used for all NMR spectral deconvolutions [40].

### 3.3. Computational NMR

Crystal structures were imported from the Crystallography Open Database (COD) [41], and structural relaxation was performed using periodic density functional theory (DFT) calculations with the Quantum Espresso [42] open source electronic structure code. Norm-conserving pseudopotentials [43] with the generalized gradient approximation in the form of Perdew, Burke, and Ernzerhof (PBE) [44] were implemented. An energy cut-off of 90 Ry and a 6x6x6 k-point matrix were used with high levels of convergence (total energy convergence threshold = 4 × 10^−8^, force convergence threshold = 2 × 10^−5^, self-consistent energy threshold = 1 × 10^−10^). Dispersion corrections were not included in the simulations. Tests indicated that the computational ^1^H-NMR isotropic chemical shifts were not impacted by the dispersion correction, changing between 0.1 to 0.5 ppm for the crystal structures studied here when it was included. For the initial identification of low-energy structures, the lattice parameters were changed iteratively to identify the lowest energy structure, which was then used as the starting point for a variable cell relaxation. Computational NMR chemical shifts were calculated using the gauge-including projector augmented wave (GIPAW) method [45], which has been proven successful at investigating ^1^H-NMR chemical shifts in proton containing MgO phases [2,23,46]. Refer to [47,48] for a more thorough description of the GIPAW method and its application to different oxide and ceramic systems. Absolute shielding tensors for the computational crystalline systems were calculated from fully converged all-electron calculations.

## 4. Conclusions

The ^1^H-MAS-NMR spectra of crystalline Mg-based phases was combined with periodic DFT NMR calculations to produce a correlation between the computed chemical shielding tensor and the experimental chemical shift to be used for referencing hydroxylated and hydrated Mg materials. This correlation was used to predict ^1^H-NMR chemical shifts for multiple Mg- and Na-based proton containing phases that have been proposed in literature, as a demonstration that computational NMR methods provide a powerful tool for the assignment of solid state ^1^H-NMR spectra. The small ^1^H chemical shifts range for these materials and significant spectral overlap hampers the identification of unique species in complex phases, but the development of very high-speed MAS-NMR and improved homonuclear decoupling techniques will continue to improve spectral resolution. In addition, the introduction of 2D heteronuclear NMR correlation experiments (^1^H-^17^O, ^1^H-^23^Na etc.) would also prove beneficial in separating different proton species in complex proton containing Mg phases and is being pursued.

## Figures and Tables

**Table 1:** ^1^H-NMR chemical shifts (δ), relative concentration (%), and computational chemical shielding (σ) of proton environments in select Mg-containing materials. Reference compounds shown in bold.

System	^1^H-NMR (ppm) [Experimental]	Conc. (%)	Average ^1^H-NMR [Computational]		
			σ (ppm)	δ (ppm) ^a^	COD ID ^c,d^
**Mg(OH)_2_**	**0.05**	93.2%	**30.75**	**−0.31**	1000054(ND)
	4.9 ^b^	6.8%			
	−0.5 to 0.0 ± 2 Refs. [34,35,36]				
**Mg(NO_3_)_2_·6H_2_O**	4.5	4.6%	**25.1 ± 0.5**	**4.6 ± 0.4**	9011207(XD)
	**4.0**	95.4%			
**MgCl_2_·6H_2_O**	4.5	2.0%	**24.0 ± 0.3**	**5.1 ± 0.2**	9011352(ND)
	**4.3**	98.0%			
**MgSO_4_·7H_2_O**	**5.0**	97.1%	**23.4 ± 0.5**	**5.55 ± 0.4**	9010743(ND)
	4.4 ^b^	2.9%			
**Mg_7_Si_4_O_14_(OH)_2_**	**3.9** Ref. [30]		**26.75 ± 0.04**	**2.76 ± 0.03**	1534957(M)
	**4.1** Ref. [30]				
**Mg_5_Si_2_O_8_(OH)_2_**	**1.1** Ref. [25]		**29.9 ± 0.3**	**0.3 ± 0.2**	9016666(XD)
MgBr_2_·6H_2_O	4.1	100%			
hydromagnesite [4MgCO_3_·Mg(OH)_2_·4H_2_O]	−1.4	10.6%			
	−1.0	8.5%			
	1.1	0.1%			
	5.5	48.3%			
	7.0	31.6%			
	16.9	0.9%			
artinite [MgCO_3_·Mg(OH)_2_·3H_2_O]	−1.6	1.8%			
	−1.3	3.3%			
	−0.4	0.6%			
	−0.1	16.9%			
	0.8	0.1%			
	5.2	39.8%			
	7.0	37.5%			
dypingite [4MgCO_3_·Mg(OH)_2_·5H_2_O]	−1.4	13.2%			
	−1.1	5.1%			
	0.7	0.2%			
	1.1	1.0%			
	5.5	52.9%			
	7.0	27.1%			
	16.9	0.5%			
**NaOH**	**−** **3.2**	72.9%	**34.5**	**−** **3.1**	2310820(XD)
	0.8 ^b^	20.5%			
	4.8 ^b^	3.0%			
	8.6 ^b^	3.5%			

^a^ Chemical shift referenced using Equation (3). ^b^ Assigned to residual surface adsorbed water. ^c^ COD ID = Crystallography Open Database ID number, ^d^ ND = neutron diffraction, XD = X-Ray diffraction, M = Model.

**Table 2:** Computational ^1^H-NMR chemical shieldings (σ) and chemical shifts (δ) for 17 Mg-O-H, Mg-Cl-O-H, and Na-O-H containing compounds.

System	^1^H-NMR [Computational]		COD ID ^b^
	σ (ppm)	δ (ppm) ^a^	
**MgO Hydroxides**			
Mg_5_O_4_(OH)_2_	30.50	0.01	1531159
Mg_4_O_3_(OH)_2_	29.56	0.74	1531161
Mg_3_O_2_(OH)_2_	29.53, 30.49	0.02, 0.78	1531164
Mg_2_O(OH)_2_	30.43	0.07	1531167
Mg_3_O(OH)_4_	29.90, 30.25	0.21, 0.49	1531171
Mg_5_O(OH)_8_	29.55, 29.56, 29.88, 30.25	0.21, 0.50, 0.76, 0.77	1531175
**Magnesium Chloride Hydrates**			
MgCl_2_·6H_2_O	23.25, 23.32, 23.73, 24.15, 25.01, 25.36	5.69, 5.63, 5.31, 4.98, 4.31, 4.04	9011352
MgCl_2_·4H_2_O	24.69, 24.99, 25.08, 25.12	4.30, 4.33, 4.40, 4.64	2018339
MgCl_2_·2H_2_O	24.92	4.41	2102790
MgCl_2_·H_2_O	25.44, 25.15	4.05, 4.28	2102791
**Magnesium Perchlorate**			
Mg(ClO_4_)_2_·4H_2_O	25.01, 25.02, 26.66, 26.67	3.07, 4.39	2104832
Mg(ClO_4_)_2_·2H_2_O	26.34	3.33	2104833
**Mg-Cements**			
3Mg(OH)_2_·MgCl_2_·8H_2_O	15.96, 22.51, 22.89, 23.27, 25.10, 25.66, 26.00, 26.16, 30.19, 30.32, 30.68	11.61, 6.38, 6.08, 5.78, 4.32, 3.87, 3.60, 3.47, 0.26, 0.15, −0.13	9010975
5Mg(OH)_2_·MgCl_2_·8H_2_O	20.18, 24.20, 24.49, 24.61, 28.10, 28.32, 28.55, 28.99, 29.71, 33.42	8.24, 5.03, 4.80, 4.71, 1.92, 1.75, 1.56, 1.21, 0.64, −2.32	2103035
**Hydrated NaOH (Layered)**			
NaOH·H_2_O	20.28, 20.8, 27.14	8.15, 7.78, 2.69	1532133
α-NaOH·4H_2_O	16.01, 18.15, 20.84, 21.03, 21.77, 25.32, 25.63, 27.67, 29.59	11.57, 9.86, 7.71, 7.56, 6.97, 4.14, 3.89, 2.27, 0.74	1532135
β-NaOH·4H_2_O	18.95, 21.46, 22.44, 24.13, 24.64, 26.65, 27.27, 30.33	9.29, 7.24, 6.45, 5.13, 4.69, 3.00, 2.60, 0.16	1532136

^a^ Referenced using Equation (3). ^b^ Crystallography Open Database ID number.

## Supplementary Materials

The following are available online, Figure S1: ^1^H-MAS-NMR spectra of dypingite from two different commercial sources, Figure S2: 2D DQ-SQ ^1^H-MAS-NMR correlation experiments for dypingite, Figure S3: The variation of water and hydroxyl intensity for the Mg carbonate series of minerals, Table S1: Computational ^1^H-NMR chemical shieldings (σ) and chemical shifts (δ) for 7 proton containing reference structures along with the atomic coordinates for reference minerals: brucite, nitromagnesite, epsomite, bischofite, wadsleyite, OH-chondrodite, and sodium hydroxide.
