# Supplementary material for: Computational and Experimental 1H-NMR Study of Hydrated Mg-Based Minerals
Source: Molecules. 2020 Feb 19;25(4):933. doi: 10.3390/molecules25040933 (PMC7070456; doi:10.3390/molecules25040933)
Supplement: Supplementary file 1 [file molecules-25-00933-s001.pdf]

Electronic Supplemental Information (ESI)

Computational and Experimental ^1H NMR Study of Hydrated Mg-based Minerals

Eric G. Sorte, Jessica M. Rimsza and Todd M. Alam

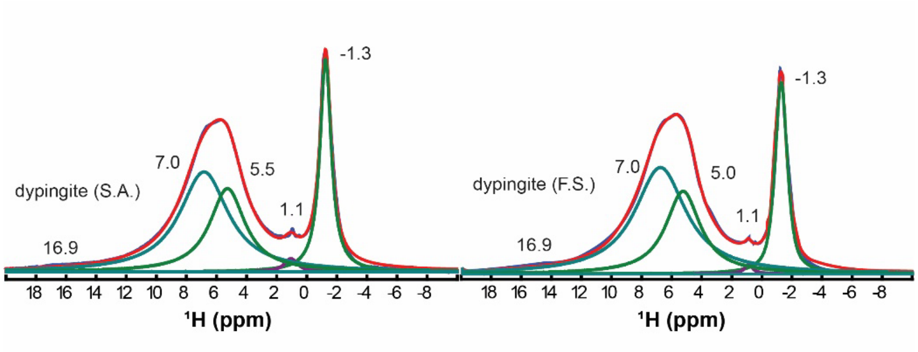

Figure S1: Deconvolution of the ^1H MAS NMR spectra for dypingite $4MgCO_3\bullet Mg(OH)_2\bullet 5H_2O$ from two different commercial sources, Fisher Scientific (F.S,.) and Sigma Aldrich (S.A.) showing almost equivalent NMR spectra, including the presence of the minor impurity resonance at $\delta = +1.1$ ppm.

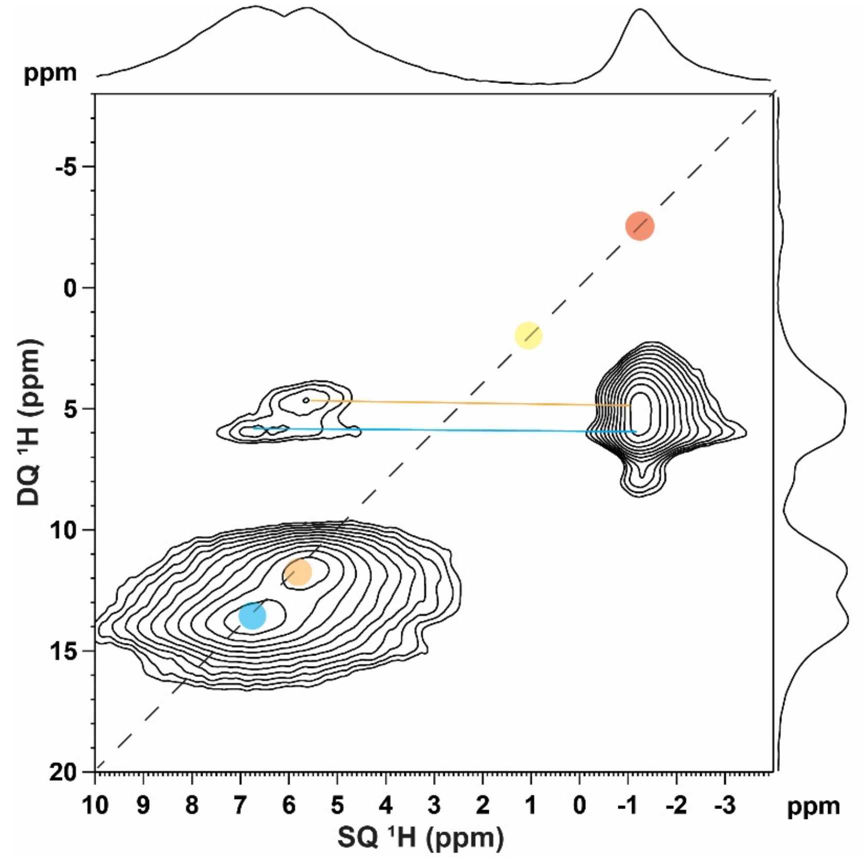

Figure S2: 2D DQ-SQ ^{1}H MAS NMR correlation experiments for dypingite, $4MgCO_3 \bullet Mg(OH)_2 \bullet 5H_2O$. The auto-correlation peaks between protons in the same environment (chemical shift) are identified by solid circles, and the colored lines identify correlation between different proton environments. These DQ-SQ correlations are very similar to those observed for hydromagnesite in Figure 2.

For dypingite two water resonances are more clearly defined at $\delta = +5.6$ ppm and + 6.8 ppm, with both environments having auto correlation peaks (solid blue and orange circles). This means that the water protons are spatially close to other water protons in the same environment (as expected). The water and hydroxyl protons ($\delta \sim -1.3$ ppm) are also correlated (solid blue and orange lines) demonstrating that the water and hydroxyl protons are spatially near each other. Note that the hydroxyl cross peak shows that the hydroxyl protons have the chemical shifts (in contrast to hydromagnesite), arguing the OH environments are equivalent. The hydroxyl protons ($\delta \sim -1.3$ ppm) show a weak autocorrelation peak (solid red circle) showing that these hydroxyl protons have limited spatial interactions near other hydroxyl protons.

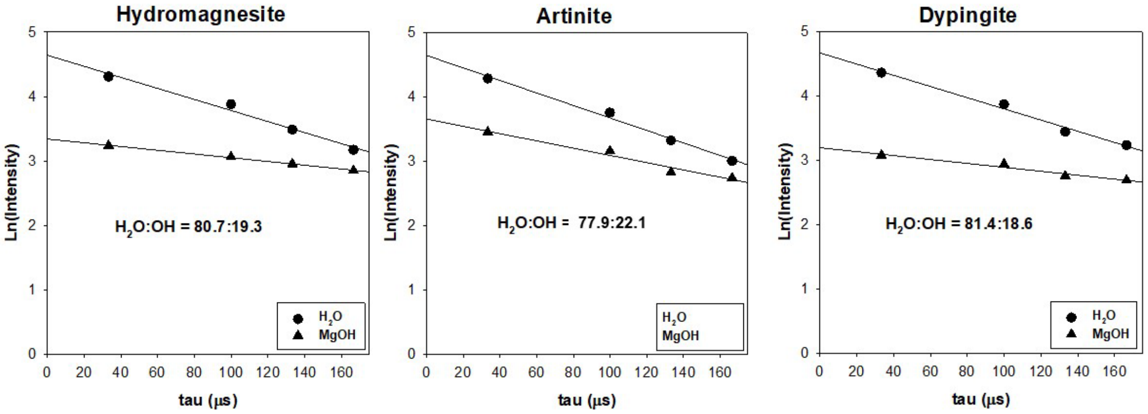

Figure S3: The variation of water and hydroxyl intensity for the Mg carbonate series of minerals as a function of rotor-synchronized echo delay τ.

The observed NMR signal will decay as a function of the spin-spin relaxation time T_2 and the echo spacing times using $S(\tau)=S(0)\exp[-2\tau/T_2]$. Quantitative signal intensities were obtained by extrapolating back to $\tau = 0$ to give $S(0)$. The relative ratios for the water and hydroxyl protons are provided in Figure S3.

Table S1: Computational ^{1}H NMR chemical shieldings (σ) and chemical shifts (δ) for 7 proton containing reference structures.

System	^{1}H NMR [Computational] σ (ppm)	δ (ppm)[a]	COD ID[b]
$Mg(OH)_2$	30.75	-0.31	1000054
$Mg(NO_3)_2 \bullet 6H_2O$	27.30, 26.16, 25.27, 24.65, 23.60, 23.09	2.14, 2.95, 3.58, 4.02, 4.76, 5.13	9011207
$MgCl_2 \bullet 6H_2O$	25.36, 25.01, 24.15, 23.75, 23.33, 23.25	3.51, 3.76, 4.37, 4.67, 4.96, 5.01	9011352
$MgSO_4 \bullet 7H_2O$	27.73, 25.56, 25.47, 24.93, 24.45, 23.81, 23.57, 23.14, 22.94, 22.17, 22.15, 21.42, 20.90, 19.89	1.83, 3.37, 3.44, 3.82, 4.16, 4.61, 4.79, 5.09, 5.23, 5.78, 5.79, 6.31, 6.68, 7.40	9010743
$Mg_7Si_4O_{14}(OH)_2$	26.80, 26.70	2.73, 2.80	1534957
$Mg_5Si_2O_8(OH)_2$	29.63, 30.25	0.75, 0.31	9016666
NaOH	34.5	-3.10	231820

[a] Referenced using Equation (3). [b] Crystallography Open Database ID number

Crystallographic Information

Structural snapshots, atomic positions, and lattice vectors for crystalline structures used to reference ^{1}H NMR chemical shifts. All units are in angstrom.

Chemical Formula	$Mg(OH)_2$	$MgNO_3$-6H_2O	$MgSO_4$-7H_2O
Structure Number	1000054	9011207	9010743
Colors	Mg (green), O (red), H (white)	Mg (green), O (red), H (white), N (blue)	Mg (green), O (red), H (white), S (yellow)
Structure	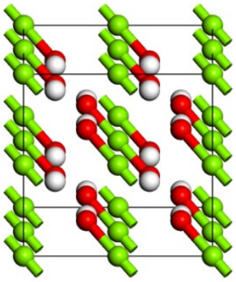	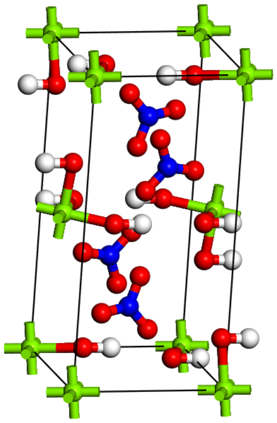	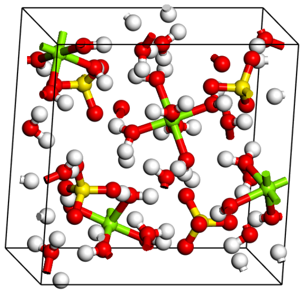

Structure: $Mg(OH)_2$
COD: 1000054

Lattice Vectors (a = 7.133Å)

0.607936791	-0.536354646	0.360739544
-0.164258020	0.792512691	0.363784828
-0.949510379	-0.553526409	0.777155142

Atomic Positions

Mg	0.000000000	0.000000000	0.000000000
Mg	0.500000000	0.500000000	0.000000000
Mg	0.500000000	0.000000000	0.000000000
Mg	0.000000000	0.500000000	0.500000000
Mg	0.000000000	0.000000000	0.500000000
Mg	0.500000000	0.500000000	0.500000000
Mg	0.500000000	-0.000000000	0.500000000

Mg	0.000000000	0.500000000	0.000000000
O	0.166687060	0.333374191	0.107947150
O	0.333374171	0.166687106	0.392052277
O	0.666625798	0.833312959	0.107946390
O	0.666668935	0.333331006	0.107963755
O	0.166688648	0.833311380	0.607952315
O	0.166687088	0.333374179	0.607947780
O	0.666625829	0.833312894	0.607947723
O	0.666669153	0.333330831	0.607964255
O	0.166688498	0.833311555	0.107951717
O	0.833312912	0.666625821	0.392052220
O	0.833311352	0.166688620	0.392047685
O	0.333331065	0.666668994	0.892036245
O	0.333374202	0.166687041	0.892053610
O	0.833312940	0.666625809	0.892052850
O	0.833311502	0.166688445	0.892048283
O	0.333330847	0.666669169	0.392035745
H	0.166679755	0.333368724	0.210206145
H	0.333369115	0.166679395	0.289793054
H	0.666631184	0.833320344	0.210206896
H	0.666671551	0.333328347	0.210222161
H	0.166686963	0.833313035	0.710211271
H	0.166679398	0.333369117	0.710206878
H	0.666630885	0.833320605	0.710206946
H	0.666671866	0.333328138	0.710223235
H	0.166686755	0.833313348	0.210210255
H	0.833320602	0.666630883	0.289793122
H	0.833313037	0.166686965	0.289788729
H	0.333328449	0.666671653	0.789777839
H	0.333368816	0.166679656	0.789793104
H	0.833320245	0.666631276	0.789793855
H	0.833313245	0.166686652	0.789789745
H	0.333328134	0.666671862	0.289776765

Structure: $MgNO_3$-$6H_2O$
COD: 9011207

Lattice Vectors (a = 8.106Å)

0.777851093	0.000037194	-0.016627512
-0.000276205	1.555905356	0.000144625
-0.017326890	0.000183832	0.846847324

Atomic Positions

N	0.474974911	0.202575592	0.555544799
N	0.525025089	0.797424408	0.444455201
N	0.475080917	0.297359746	0.055281651
N	0.524919083	0.702640254	0.944718349
O	0.598591280	0.122713458	0.546440574
O	0.401408720	0.877286542	0.453559426
O	0.598563392	0.377296723	0.046241561
O	0.401436608	0.622703277	0.953758439
O	0.279021017	0.190393469	0.595659717
O	0.720978983	0.809606531	0.404340283
O	0.279347981	0.309363129	0.096323228
O	0.720652019	0.690636871	0.903676772
O	0.545498820	0.296787498	0.524421599
O	0.454501180	0.703212502	0.475578401
O	0.545496286	0.203254597	0.023179936
O	0.454503714	0.796745403	0.976820064
O	0.965760494	0.338588041	0.469645647
O	0.034239506	0.661411959	0.530354353
O	0.966025274	0.161410923	0.969319129
O	0.033974726	0.838589077	0.030680871
O	0.295293613	0.475507534	0.640369471
O	0.704706387	0.524492466	0.359630529
O	0.295526320	0.024397737	0.139856513
O	0.704473680	0.975602263	0.860143487
O	0.856932900	0.498691171	0.769036501
O	0.143067100	0.501308829	0.230963499
O	0.857324985	0.001489400	0.269269631
O	0.142675015	0.998510600	0.730730369
H	0.074050674	0.287206225	0.521951354
H	0.925949326	0.712793775	0.478048646
H	0.074209878	0.212757992	1.021926940
H	0.925790122	0.787242008	-0.021926940
H	0.357577926	0.516666125	0.751652534
H	0.642422074	0.483333875	0.248347466

H	0.357674783	-0.016593790	0.251449849
H	0.642325217	1.016593790	0.748550151
H	-0.197893770	0.565400820	0.828568581
H	1.197893770	0.434599180	0.171431419
H	-0.197523386	-0.065188254	0.329010054
H	1.197523386	1.065188254	0.670989946
H	0.821498471	0.308568119	0.491188926
H	0.178501529	0.691431881	0.508811074
H	0.821725909	0.191475010	0.990376806
H	0.178274091	0.808524990	0.009623194
H	0.396970431	0.418885055	0.605975059
H	0.603029569	0.581114945	0.394024941
H	0.397178932	0.081026950	0.105313252
H	0.602821068	0.918973050	0.894686748
H	0.802857735	0.439350722	0.846370440
H	0.197142265	0.560649278	0.153629560
H	0.803260096	0.060859950	0.346438533
H	0.196739904	-0.060859950	0.653561467
Mg	0.000000000	0.500000000	0.500000000
Mg	0.000000000	0.000000000	0.000000000

Structure: $MgSO_4$-$7H_2O$

COD: 9010743

Lattice Vectors (a = 10.584Å)

0.701531746	-0.117811453	-0.017663436
-0.105624771	0.614610314	-0.103989285
-0.010947944	-0.094328634	0.573909685

Atomic Positions

Mg	0.387522492	0.116149063	0.051306280
S	0.870175263	0.038541561	0.513630169
O	0.678209766	-0.120679057	0.388979497
O	1.001632055	-0.092087125	0.491401596
O	0.854795554	0.138496626	0.768148055
O	0.938392457	0.222328197	0.415499225
O	0.179603500	0.275756900	0.089285490
O	0.600479974	0.410986894	0.284795672
O	0.494916224	0.214964160	0.771042144
O	0.592144260	-0.043581356	0.009375952
O	0.175764115	0.835971002	0.829888887
O	0.320037649	-0.009277384	0.309123590
O	0.356093621	0.535084770	0.825487824
H	0.114201378	0.274146503	0.231114841
H	0.069297273	0.217630342	0.963653569
H	0.626628406	0.576536920	0.323927923
H	0.721334182	0.395775322	0.327873396
H	0.464799604	0.355458316	0.775265210
H	0.633878758	0.246310751	0.776531096
H	0.641357024	-0.061035666	0.155650503
H	0.700810436	1.006263305	-0.078041012
H	0.199408842	0.690945139	0.774391571
H	0.100900228	0.846828394	0.699889477
H	0.187373227	-0.049570310	0.351736707
H	0.401254688	0.055021861	0.461846699
H	0.299175378	0.506055668	0.962692180
H	0.455313647	0.691279203	-0.111896547

Chemical Formula	$MgCl_2$-6H_2O	$Mg_7Si_4O_{14}(OH)_2$	NaOH
Structure Number	9011352	1534957	2310820
Colors	Mg (green), O (red), H (white), Cl (blue)	Mg (green), O (red), H (white), Si (yellow)	O (red), H (white), Na (purple)
Structure	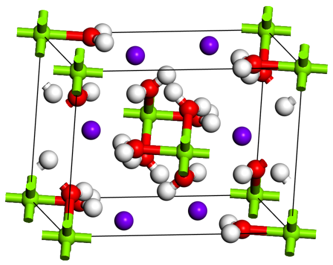	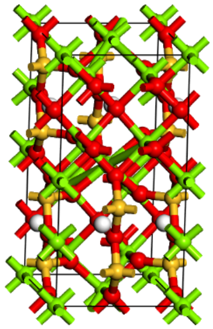	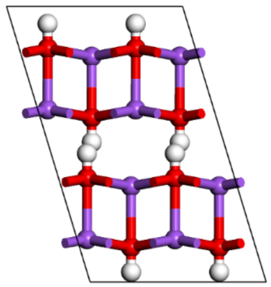

Structure: $MgCl_2$•$6H_2O$
COD: 9011352

Lattice Vectors (a = 10.361 Å)

1.026124106	-0.146692689	-0.125657898
-0.111264377	0.686449843	-0.132298323
-0.063114106	-0.096643160	0.676025616

Atomic Positions

Mg	0.000000000	0.000000000	0.000000000
Mg	0.500000000	-0.000000000	0.000000000
Cl	0.222622857	0.307986085	0.694201136
Cl	0.277378793	0.692014702	0.305799128
Cl	0.722621207	0.307985298	0.694200872
Cl	0.777377143	0.692013915	0.305798864
O	0.182127978	0.224890359	0.226280731
O	0.317877721	0.775112631	0.773723165
O	0.682122279	0.224887369	0.226276835
O	0.817872022	0.775109641	0.773719269
O	0.393293635	0.078695793	0.205110272
O	0.106706691	0.921304860	0.794890952
O	0.478549109	0.217264665	0.878330151
O	0.021450781	0.782735304	0.121669692
O	0.893293309	0.078695140	0.205109048
O	0.606706365	0.921304207	0.794889728
O	0.978549219	0.217264696	0.878330308
O	0.521450891	0.782735335	0.121669849
H	0.185093139	0.241959552	0.374103110
H	0.314904569	0.758037754	0.625897922
H	0.685095431	0.241962246	0.374102078
H	0.814906861	0.758040448	0.625896890
H	0.204923927	0.368935171	0.230330255
H	0.295074512	0.631065522	0.769666780
H	0.704925488	0.368934478	0.230333220
H	0.795076073	0.631064829	0.769669745
H	0.324030376	0.146456227	0.201290124
H	0.175968940	0.853543203	0.798708671
H	0.397905903	0.249969442	0.824617530
H	0.102094063	0.750030411	0.175382543
H	0.824031060	0.146456797	0.201291329
H	0.675969624	0.853543773	0.798709876
H	0.897905937	0.249969589	0.824617457
H	0.602094097	0.750030558	0.175382470
H	0.360815385	-0.026152523	0.264370024
H	0.139184518	1.026152440	0.735629656

H	0.548039723	0.272128152	0.815505535
H	-0.048039527	0.727871924	0.184494759
H	0.860815482	-0.026152440	0.264370344
H	0.639184615	1.026152523	0.735629976
H	1.048039527	0.272128076	0.815505241
H	0.451960277	0.727871848	0.184494465

Structure: $Mg_7Si_4O_{14}(OH)_2$
COD: 1534957

Lattice Vectors (a = 5.821Å)

0.995896231	0.000000000	0.000000000
0.000000000	1.983482546	0.000000000
0.000000000	0.000000000	1.420644551

Atomic Positions

Si	0.500000000	0.623497367	0.110954028
Si	0.500000000	0.876502633	0.110954028
Si	0.500000000	0.123497367	0.889045972
Si	0.500000000	0.376502633	0.889045972
Si	-0.000000000	0.118524402	0.607642023
Si	0.000000000	0.381475598	0.607642023
Si	-0.000000000	0.618524402	0.392357977
Si	0.000000000	0.881475598	0.392357977
Mg	0.500000000	0.500000000	0.500000000
Mg	0.500000000	0.000000000	0.500000000
Mg	0.000000000	0.000000000	0.000000000
Mg	0.000000000	0.500000000	0.000000000
Mg	0.253528512	0.114446218	0.251480454
Mg	0.746471488	0.114446218	0.251480454
Mg	0.253528512	0.385553782	0.251480454
Mg	0.746471488	0.385553782	0.251480454
Mg	0.746471488	0.614446218	0.748519546
Mg	0.746471488	0.885553782	0.748519546
Mg	0.253528512	0.885553782	0.748519546
Mg	0.253528512	0.614446218	0.748519546
Mg	0.000000000	0.250000000	0.952551220
Mg	0.000000000	0.750000000	0.047448780
O	0.258380179	0.124461182	1.001556567
O	0.741619821	0.124461182	1.001556567
O	0.258380179	0.375538818	1.001556567
O	0.741619821	0.375538818	1.001556567
O	0.741619821	0.624461182	-0.001556567
O	0.741619821	0.875538818	-0.001556567
O	0.258380179	0.875538818	-0.001556567
O	0.258380179	0.624461182	-0.001556567
O	0.500000000	0.487490620	0.761724534
O	0.500000000	0.012509380	0.761724534
O	0.500000000	0.987490620	0.238275466
O	0.500000000	0.512509380	0.238275466
O	0.000000000	0.250000000	0.708568430
O	0.000000000	0.750000000	0.291431570
O	0.000000000	0.250000000	0.238792272

O	0.000000000	0.750000000	0.761207728
O	0.500000000	0.750000000	0.718888586
O	0.500000000	0.250000000	0.281111414
O	0.500000000	0.750000000	0.203909981
O	0.500000000	0.250000000	0.796090019
O	-0.000000000	0.985209948	0.248993491
O	0.000000000	0.514790052	0.248993491
O	-0.000000000	0.485209948	0.751006509
O	0.000000000	0.014790052	0.751006509
O	0.769650955	0.617227541	0.505676928
O	0.230349045	0.617227541	0.505676928
O	0.769650955	0.882772459	0.505676928
O	0.230349045	0.882772459	0.505676928
O	0.230349045	0.117227541	0.494323072
O	0.230349045	0.382772459	0.494323072
O	0.769650955	0.382772459	0.494323072
O	0.769650955	0.117227541	0.494323072
H	0.500000000	0.750000000	0.597635151
H	0.500000000	0.250000000	0.402364849
H	0.000000000	0.250000000	0.359904809
H	0.000000000	0.750000000	0.640095191

Structure: NaOH
COD: 2310820

Lattice Vectors (a = 3.86Å)

0.913916674	0.000000000	0.031991669
0.000000000	0.909238643	0.000000000
-0.512181921	0.000000000	1.494038802

Atomic Positions

Na	0.158614276	0.250000000	0.818922215
Na	0.841385724	0.750000000	0.181077785
O	0.370101267	0.250000000	0.237183599
O	0.629898733	0.750000000	0.762816401
H	0.452879217	0.250000000	0.405654422
H	0.547120783	0.750000000	0.594345578

Chemical Formula	**$Mg_5Si_2O_8(OH)_2$**
Structure Number	9016666
Colors	Mg (green), O (red), H (white), Si (yellow)
Structure	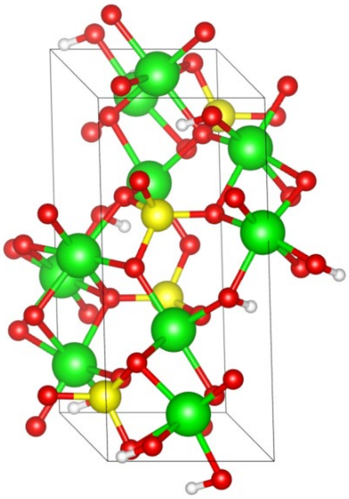

Structure: $Mg_5Si_2O_8(OH)_2$
COD: 9016666

Lattice Vectors (a = 4.76Å)

1.001482750	0.000000000	0.000000000
0.000000000	2.182393828	0.002174687
0.000000000	-0.533720894	1.579503117

Atomic Positions

H	1.083996935	1.014087873	1.012226743
H	0.416003065	0.514087873	1.012226743
H	0.558707886	0.898097012	0.794377356
H	0.941292114	0.398097012	0.794377356
Si	0.077232341	0.136951918	0.699229956
Si	0.920616067	0.856574451	0.295740961
Si	0.422767659	0.636951918	0.699229956
Si	0.579383933	0.356574451	0.295740961
Mg	0.499901976	-0.001556387	0.495821128
Mg	0.000098024	0.498443613	0.495821128
Mg	0.011579530	0.172209789	0.304205834
Mg	0.994881881	0.820078066	0.685763841
Mg	0.488420470	0.672209789	0.304205834
Mg	0.505118119	0.320078066	0.685763841
Mg	0.487624651	0.886820441	0.079825929

Mg	0.512658385	0.119826095	0.926708442
Mg	0.012375349	0.386820441	0.079825929
Mg	0.987341615	0.619826095	0.926708442
O	0.773349686	0.000980266	0.293059918
O	0.224053611	0.992053950	0.698730800
O	0.726650314	0.500980266	0.293059918
O	0.275946389	0.492053950	0.698730800
O	0.731078056	0.242310729	0.125720685
O	0.272252996	0.753839373	0.868790458
O	0.768921944	0.742310729	0.125720685
O	0.227747004	0.253839373	0.868790458
O	0.226020570	0.164832790	0.524803230
O	0.771121521	0.829902510	0.470348916
O	0.273979430	0.664832790	0.524803230
O	0.728878479	0.329902510	0.470348916
O	0.262087092	0.854346367	0.294689987
O	0.735822620	0.140673372	0.700099966
O	0.237912908	0.354346367	0.294689987
O	0.764177380	0.640673372	0.700099966
O	0.244599204	0.054144886	0.090359363
O	0.714471055	0.933826503	0.879496490
O	0.255400796	0.554144886	0.090359363
O	0.785528945	0.433826503	0.879496490
